# Supplementary material for: Pyramid diffractive optical networks for unidirectional image magnification and demagnification
Source: Light Sci Appl. 2024 Jul 31;13:178. doi: 10.1038/s41377-024-01543-w (PMC11291656; doi:10.1038/s41377-024-01543-w)
Supplement: Supplementary file 1 — Supplementary Information [file 41377_2024_1543_MOESM1_ESM.pdf]

Supplementary Information for

Pyramid diffractive optical networks for unidirectional image magnification and demagnification

Bijie Bai [1,2,3,†], Xilin Yang[1,2,3,†], Tianyi Gan[1,3], Jingxi Li[1,2,3], Deniz Mengu[1,2,3], Mona Jarrahi[1,3], and Aydogan Ozcan[*,1,2,3]

[1]Electrical and Computer Engineering Department, University of California, Los Angeles, CA, 90095, USA.

[2]Bioengineering Department, University of California, Los Angeles, 90095, USA.

[3]California NanoSystems Institute (CNSI), University of California, Los Angeles, CA, USA.

[†]Equal contributing authors

[*]Correspondence: Aydogan Ozcan. Email: ozcan@ucla.edu

Contents:

Figure S1. Examples of blind testing results of six pyramid unidirectional image magnifier diffractive networks trained using different energy boost factors, $\boldsymbol{\beta}$.

Figure S2. Examples of blind testing results for pyramid unidirectional image magnifier diffractive networks trained with different numbers of layers (a-e). The table in (f) shows the quantitative results in terms of MSE, PCC, and forward energy efficiency.

Figure S3. Imaging of horizontal resolution test targets.

Figure S4. Imaging of vertical resolution test targets.

Figure S5. Slanted-edge testing.

Figure S6. Examples of blind testing results of six pyramid unidirectional image demagnifier diffractive networks trained using different energy boost factors, $\boldsymbol{\beta}$.

Figure S7. Comparison of different P-D^{2}NN architectures for unidirectional image magnification.

Figure S8. Cascaded unidirectional P-D^{2}NN architecture with end-to-end optimization.

Figure S9. Cascaded unidirectional P-D^{2}NN architecture with individual optimization.

Figure S10. Phase quantization error analysis.

Figure S11. Fabrication error analysis.

Figure S12. Schematic of the THz source and detection system.

Figure S13. A photograph of the 3D-printed diffractive network holder.

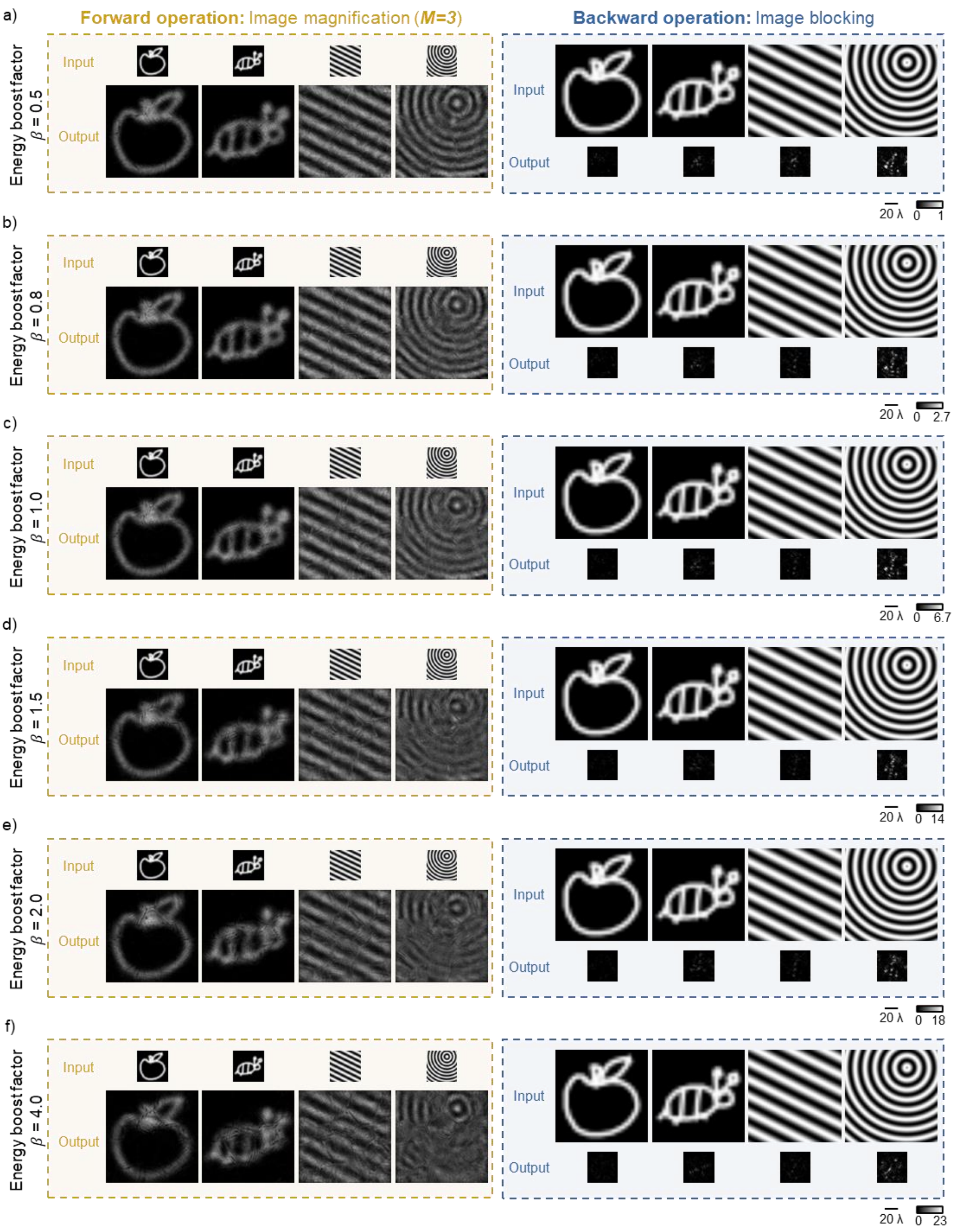

Figure S1. Examples of blind testing results of six pyramid unidirectional image magnifier diffractive networks trained using different energy boost factors, β.

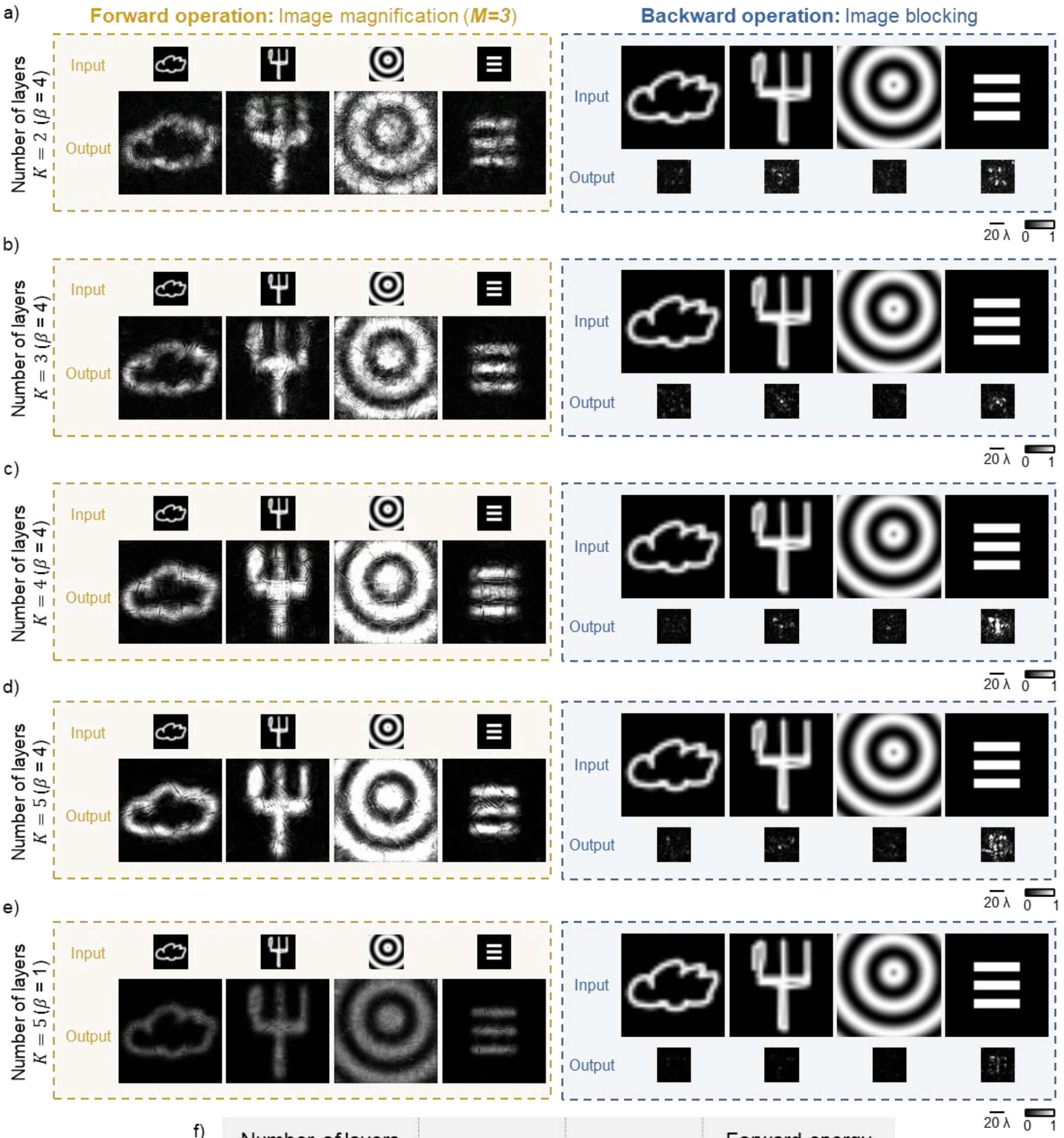

f)

Number of layers (K)	MSE	PCC	Forward energy (%)
2 (β = 4)	0.066	0.739	50.17
3 (β = 4)	0.051	0.807	50.84
4 (β = 4)	0.042	0.828	61.45
5 (β = 4)	0.033	0.867	62.91
5 (β = 1)	0.021	0.931	20.26

Figure S2. Examples of blind testing results for pyramid unidirectional image magnifier diffractive networks trained with different numbers of layers (a-e). The table in (f) shows the quantitative results in terms of MSE, PCC, and forward energy efficiency.

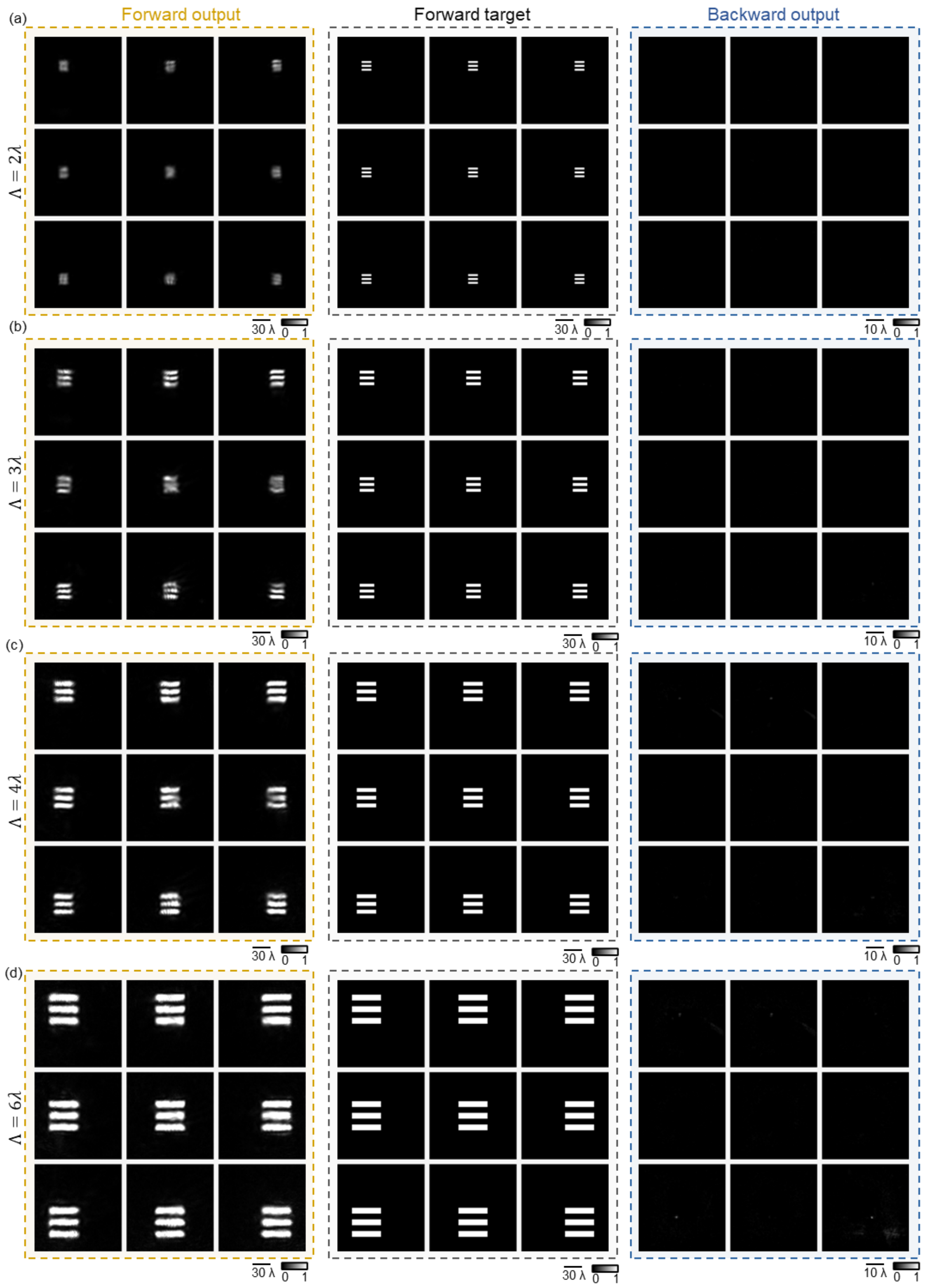

Figure S3. Imaging of horizontal resolution test targets. Four different resolution test targets (a-d) with varying grating periods (Λ, measured at the input aperture) were tested. For each resolution test target, forward output, ideal target (ground truth), and backward output images are presented. Each resolution

target was positioned at 9 different locations, arranged in a 3×3 grid. All the forward and backward output images are normalized using the same scale.

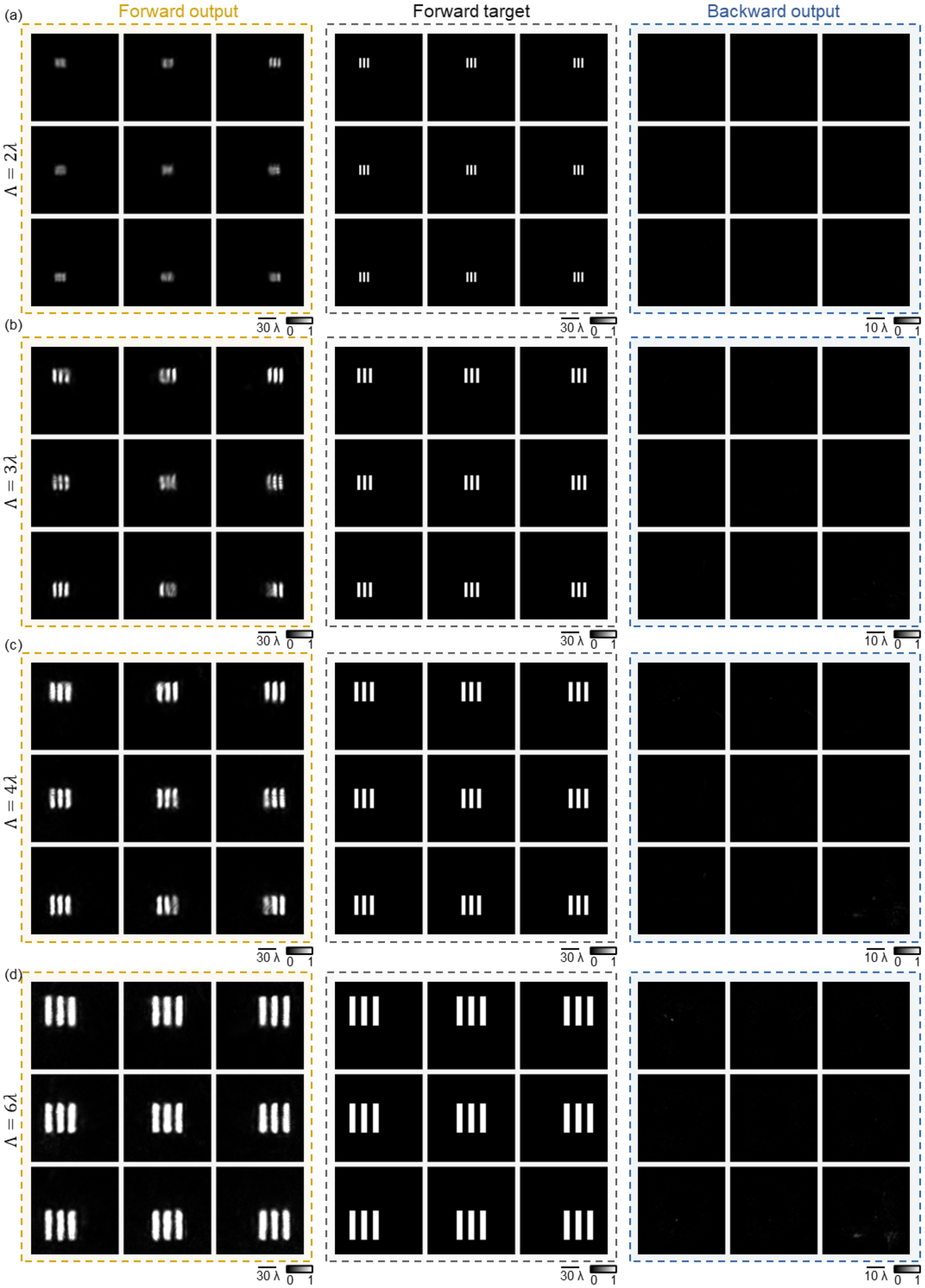

Figure S4. Imaging of vertical resolution test targets. Same as in Supplementary Figure S3, except that the resolution test targets are oriented in the vertical direction. All the forward and backward output images are normalized using the same scale.

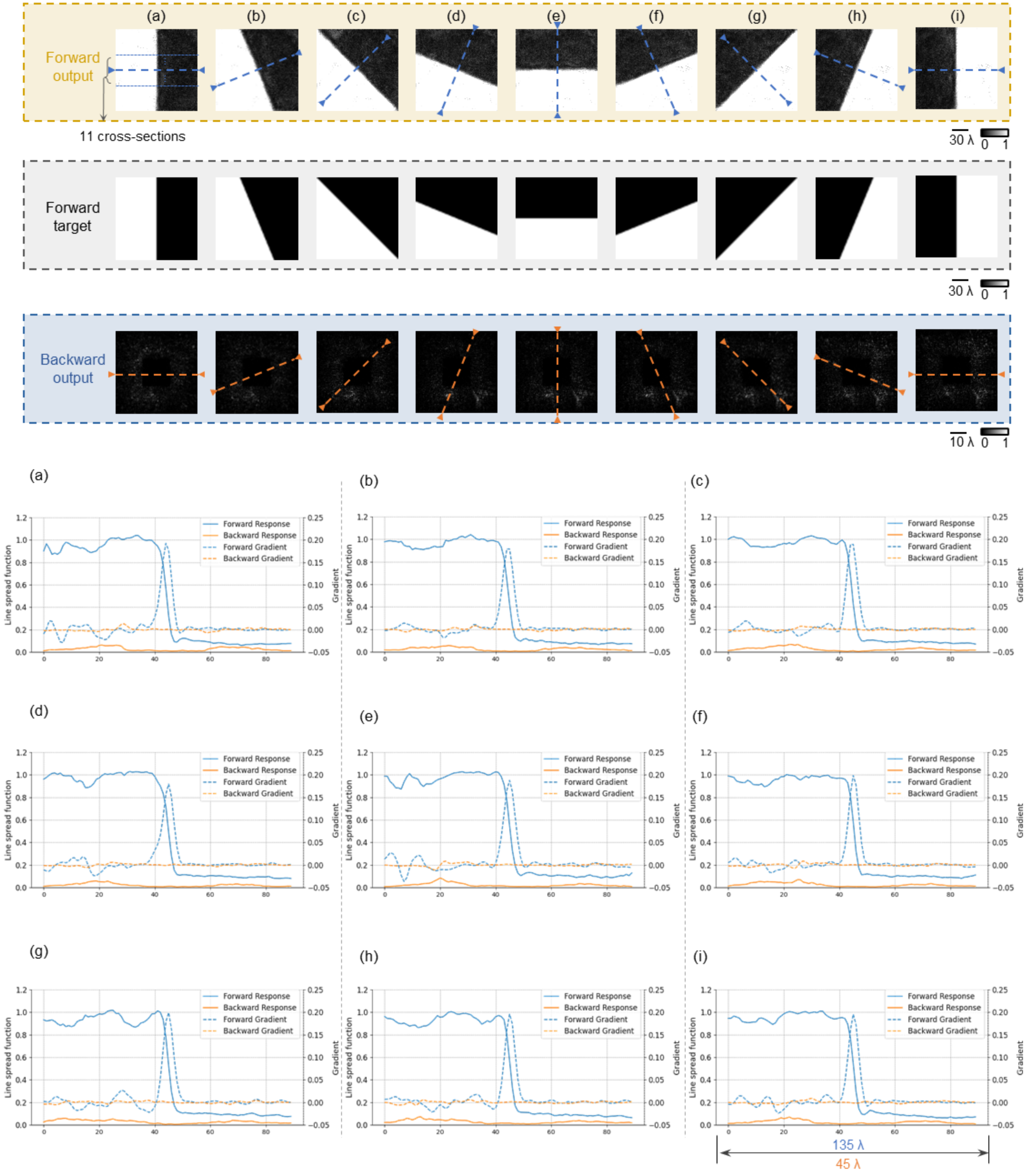

Figure S5. Slanted-edge testing. P-D^{2}NN was tested using a rotating slanted edge at nine different angles (a-i). The first three rows display the forward output, the ideal target (ground truth), and the backward output images, respectively. The blue and orange lines indicate the cross sections, which are illustrated in the plots below. All the forward and backward output images are normalized using the same scale.

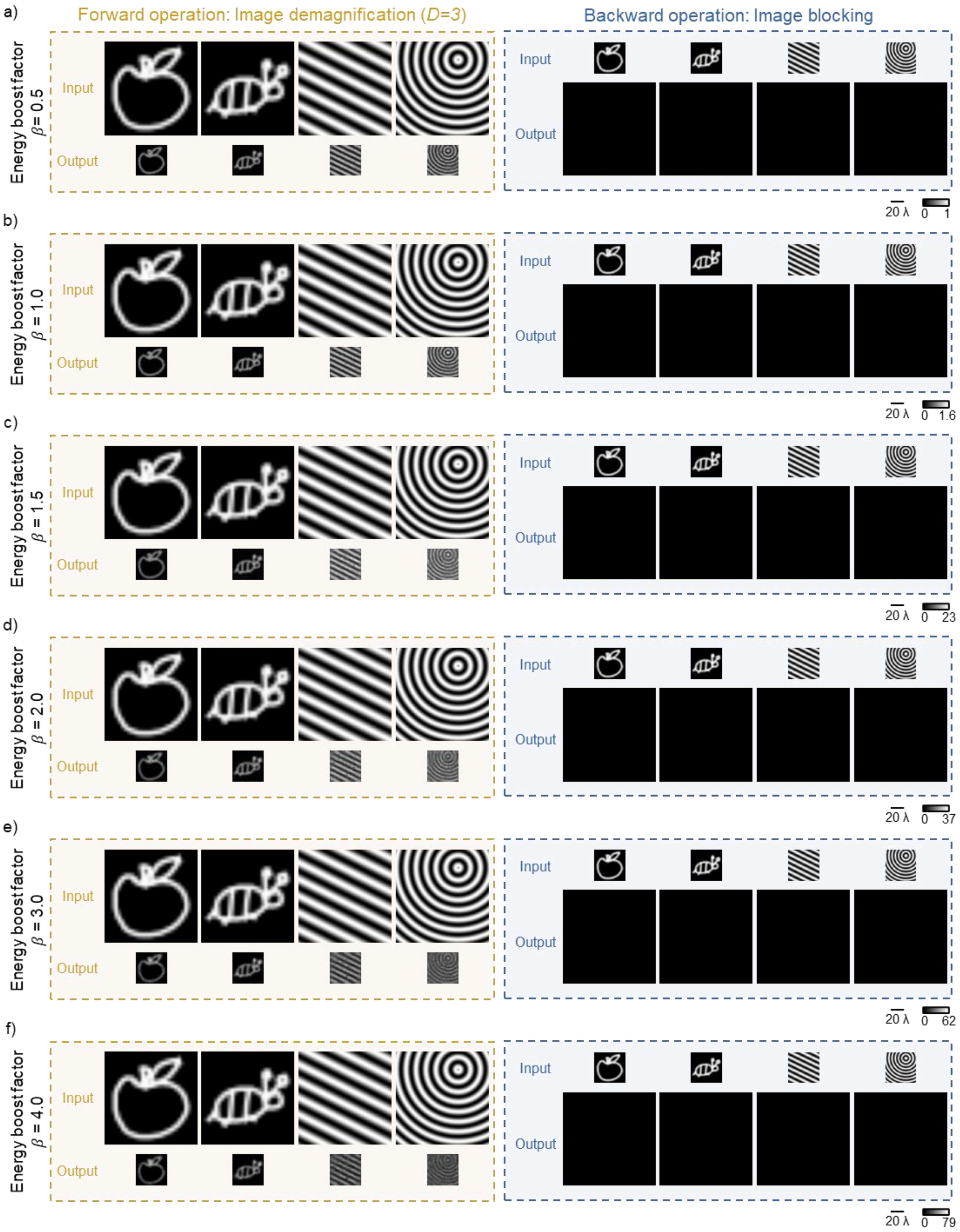

Figure S6. Examples of blind testing results of six pyramid unidirectional image demagnifier diffractive networks trained using different energy boost factors, β.

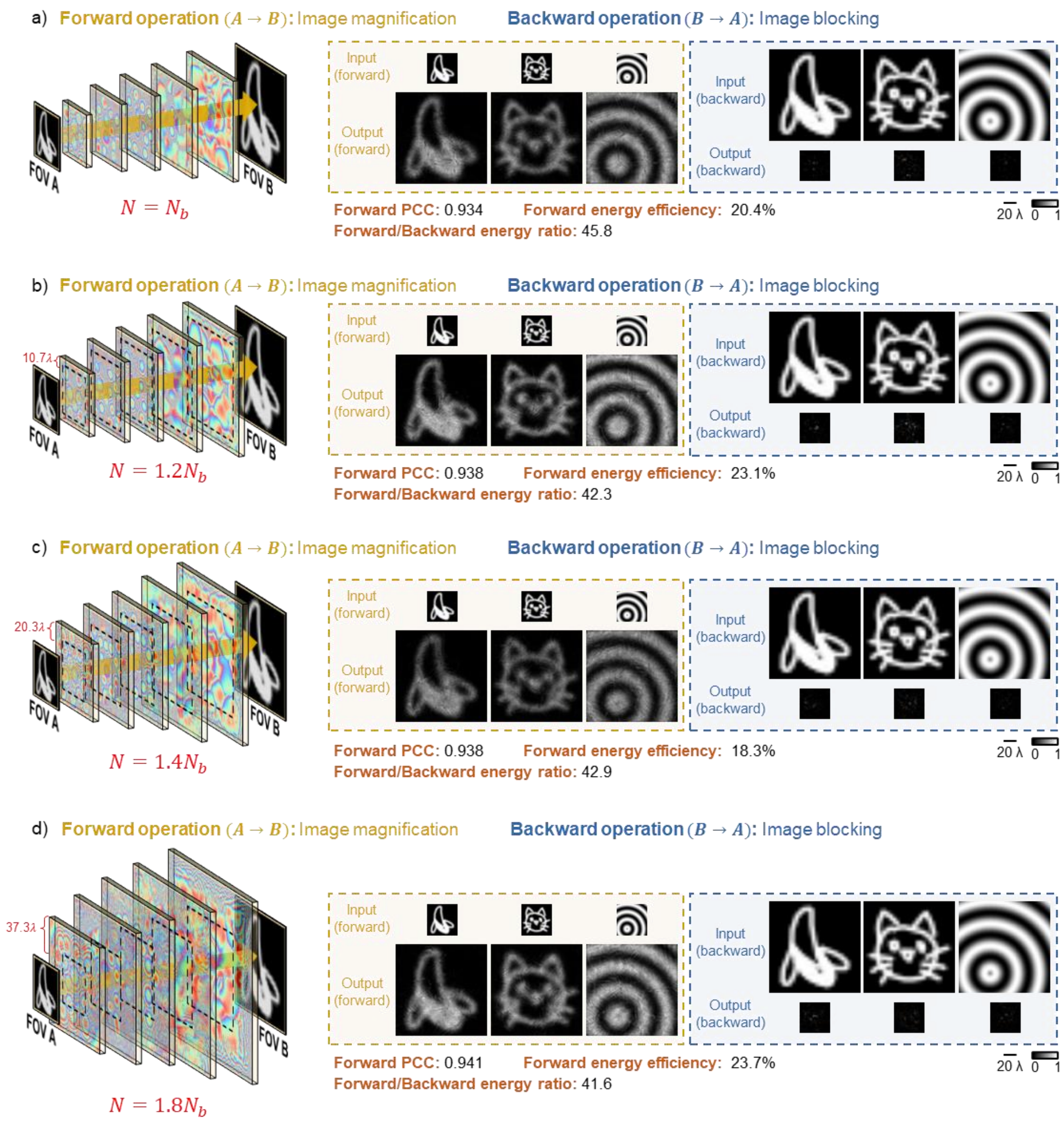

Figure S7. Comparison of different P-D^{2}NN architectures for unidirectional image magnification. (a) The design layout and blind testing results of the baseline P-D^{2}NN with $N = N_b$ trainable diffractive features, as reported in the main text Figs. 2b-c. (b-d) The design layouts and blind testing results of three distinct P-D^{2}NNs with enlarged diffractive layers, each having $N = 1.2N_b$, $N = 1.4N_b$, and $N = 1.8N_b$ trainable diffractive features, respectively.

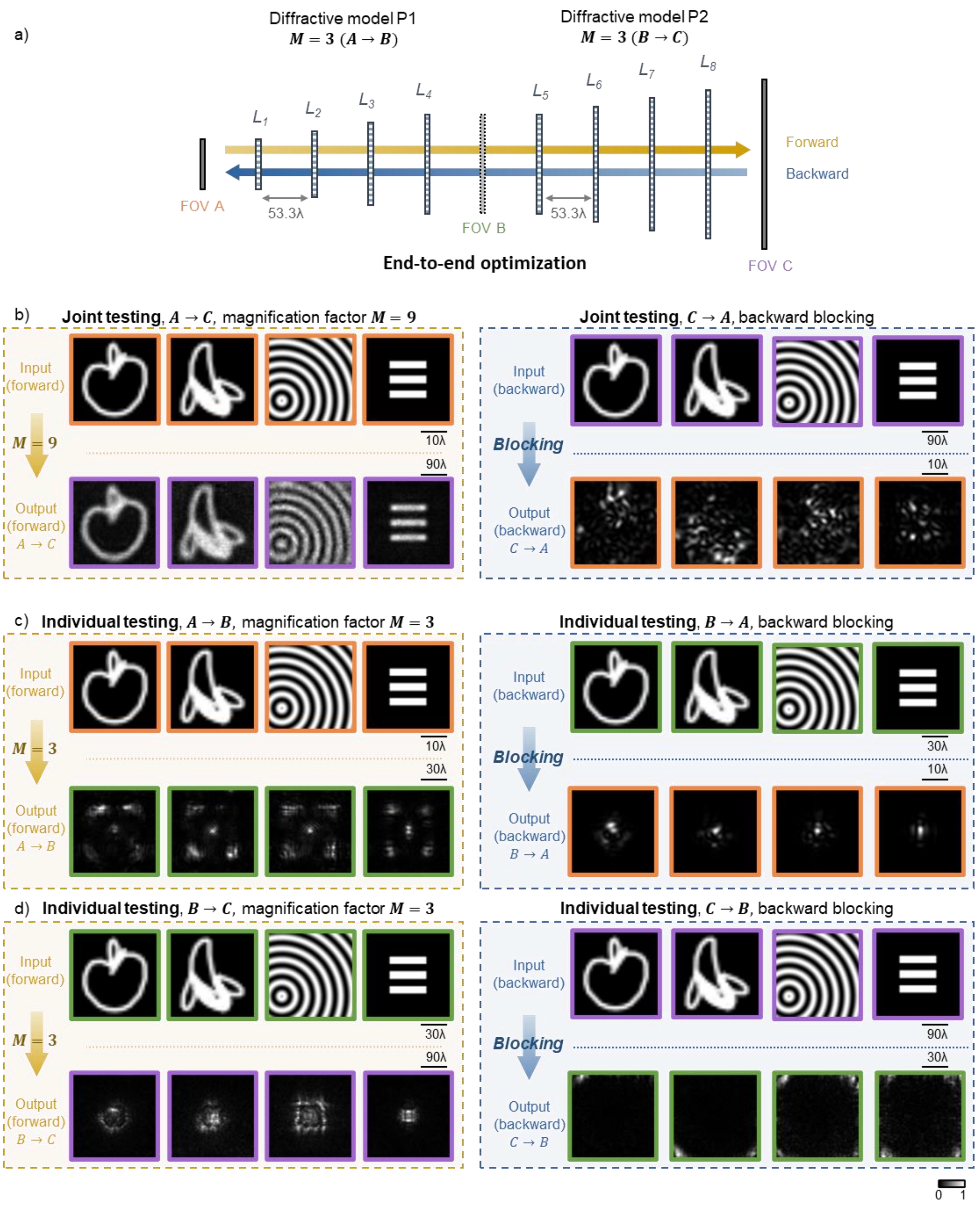

Figure S8. Cascaded unidirectional P-D²NN architecture with end-to-end optimization. (a) The cascaded diffractive structure is only optimized end-to-end, along the forward and backward paths, $A \rightarrow C$ and $C \rightarrow A$. (b) Joint testing of the cascaded diffractive network. (c) Separate testing of diffractive network P1. (d) Separate testing of diffractive network P2.

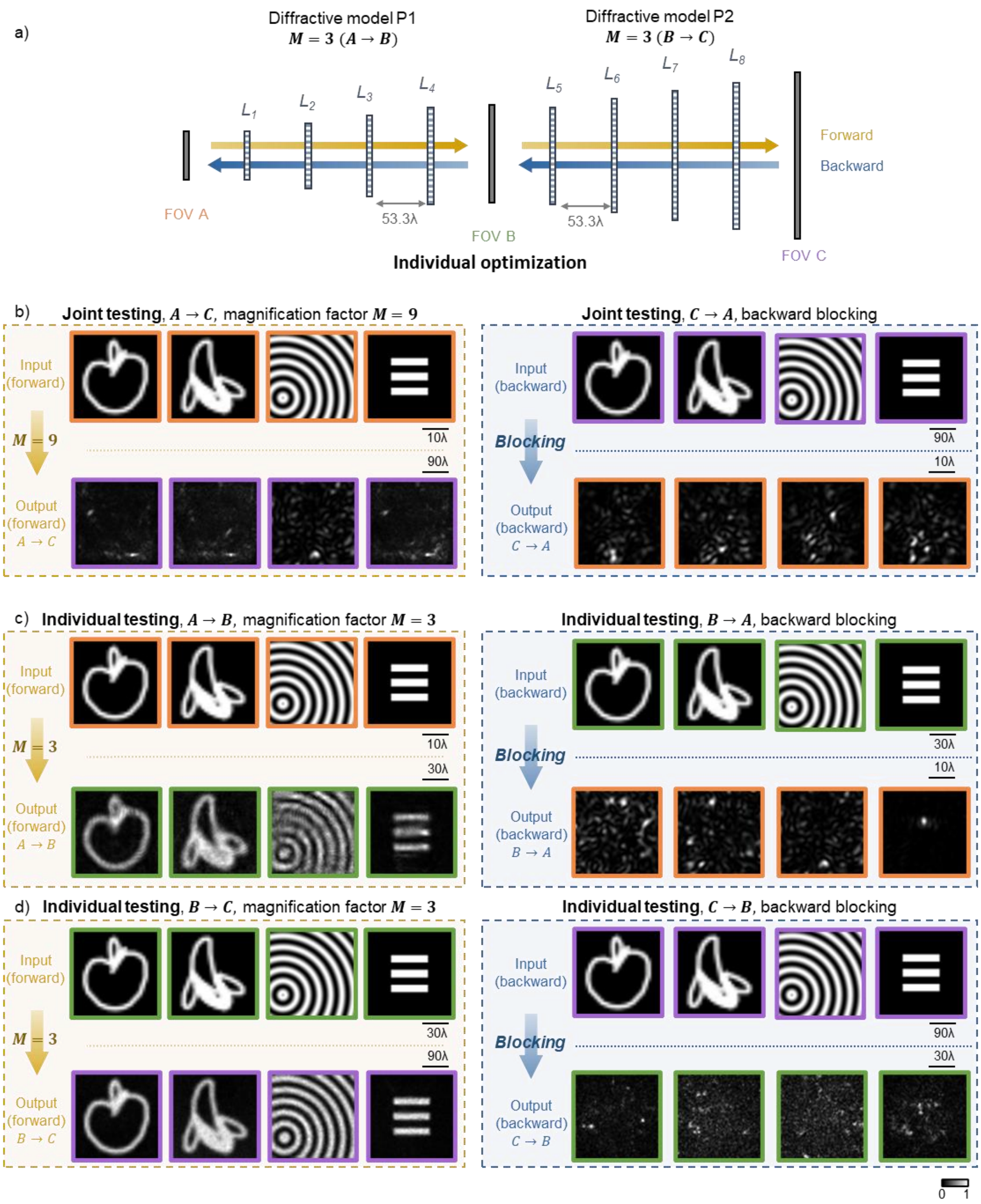

Figure S9. Cascaded unidirectional P-D^{2}NN architecture with individual optimization. (a) The cascaded diffractive structure is only optimized as two individual components (P1 and P2), without any end-to-end optimization. (b) Joint testing of the cascaded diffractive network. (c) Separate testing of diffractive network P1. (d) Separate testing of diffractive network P2.

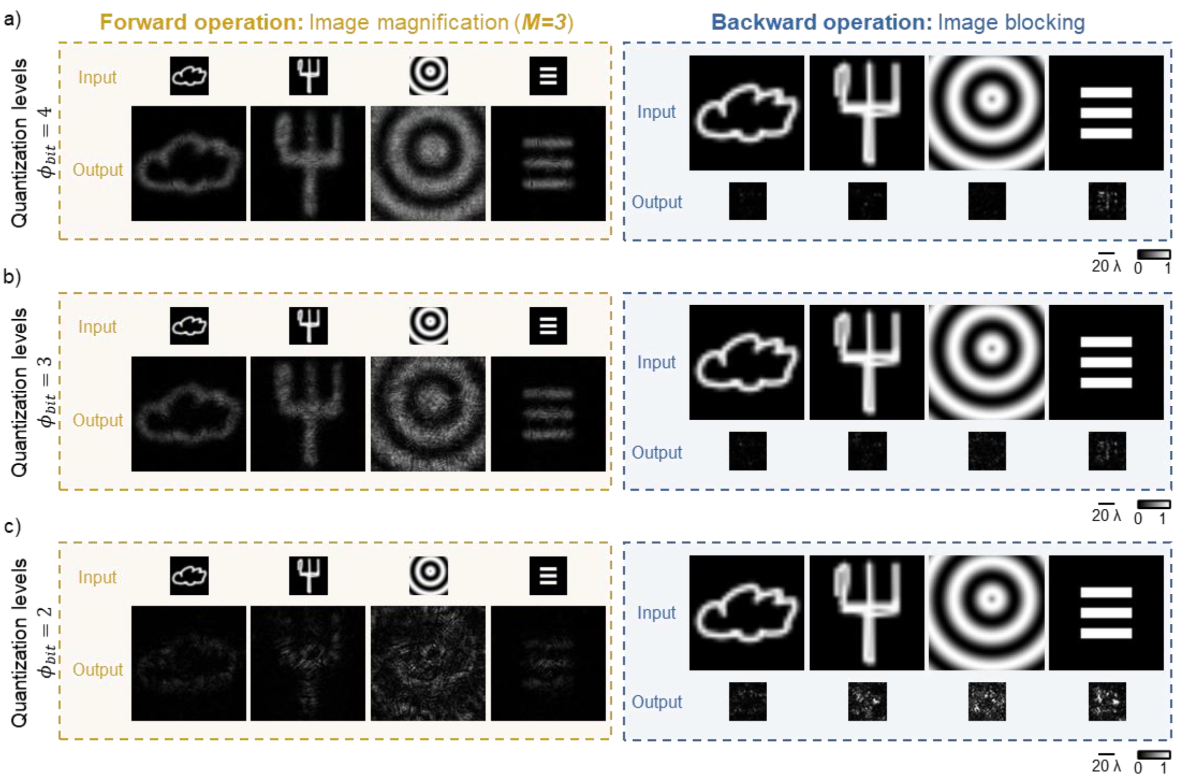

Figure S10. Phase quantization error analysis. For better visualization of the results, the images are normalized with respect to each model.

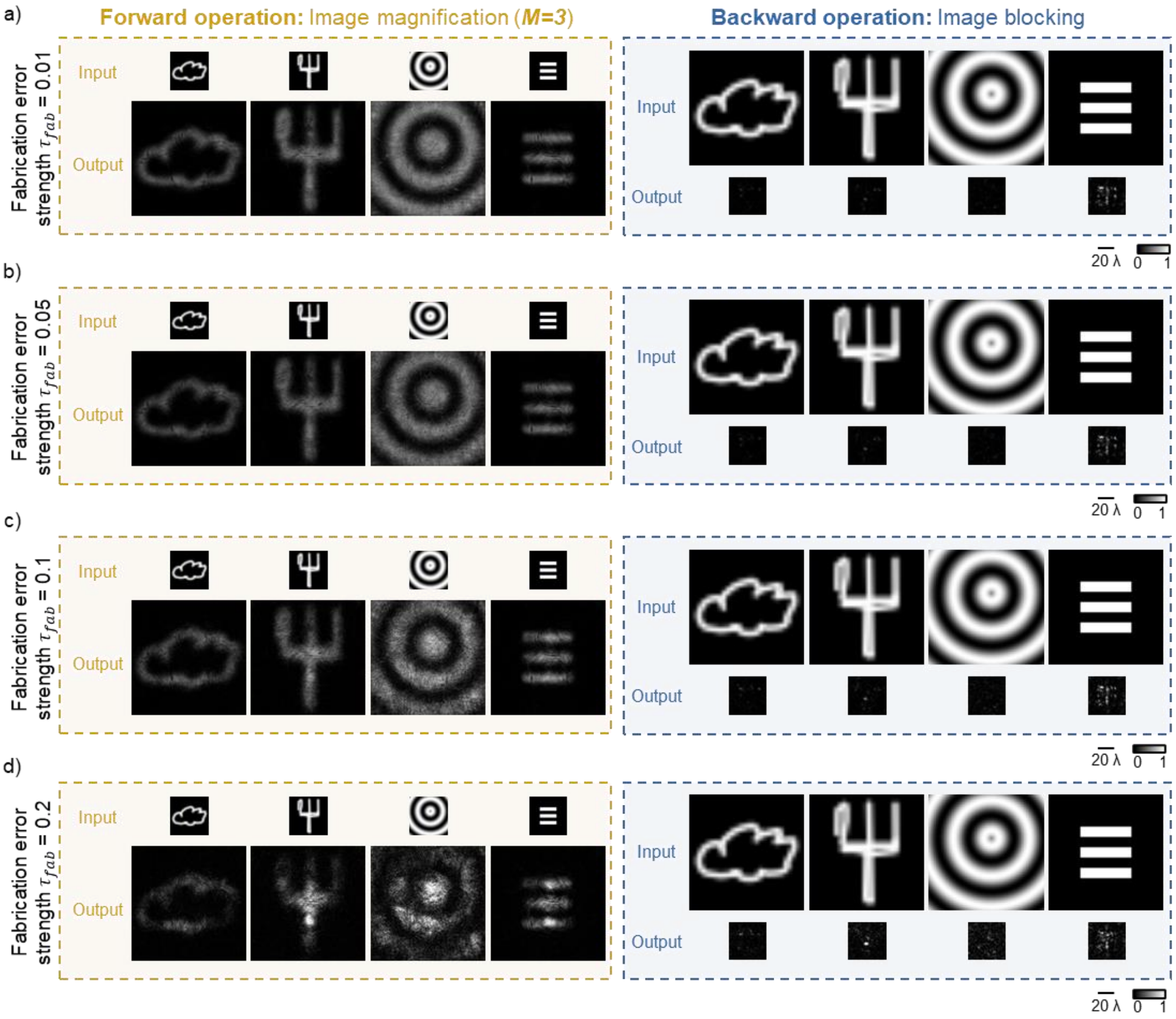

Figure S11. Fabrication error analysis. For better visualization of the results, the images are normalized with respect to each model.

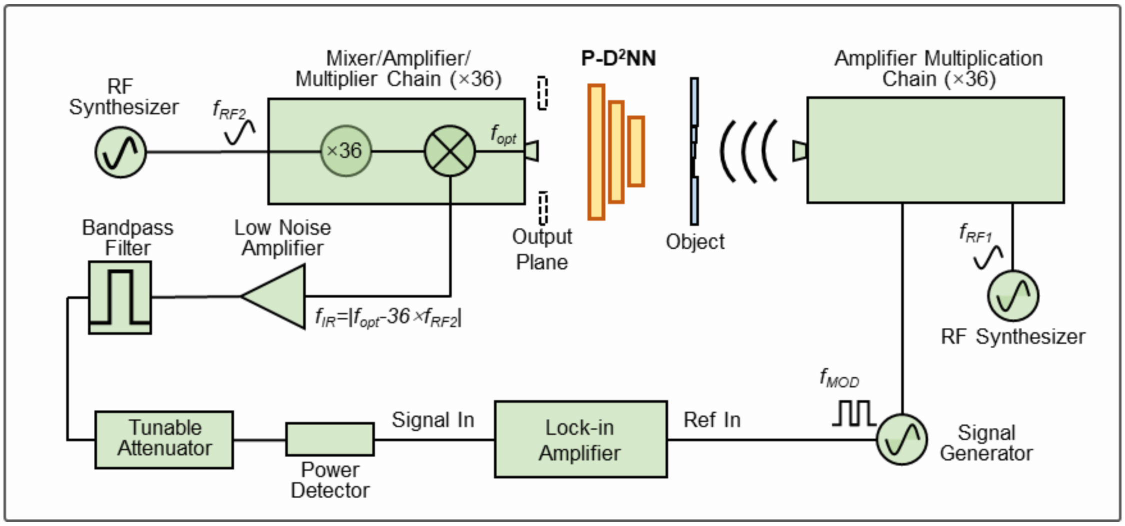

Figure S12. Schematic of the THz source and detection system.

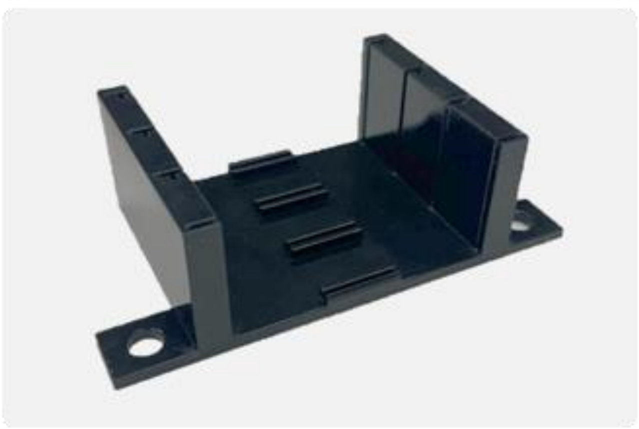

Figure S13. A photograph of the 3D-printed diffractive network holder.
